# Medical students’ perspective: project-based learning approach to increase medical student empathy

**DOI:** 10.1080/10872981.2020.1794342

**Published:** 2020-07-22

**Authors:** Simren-Jai Nathwani, Nidhi Vedd

**Affiliations:** a Biomedical Science BSc (Hons, Warwick University, Warwick, UK; b Department of Medicine & Dentistry, Barts and the London School of Medicine and Dentistry, London, UK; c Psychology iBSc (Hons, Kings College London, London, UK; d Department of Medicine, Brighton and Sussex Medical School, Brighton, UK

**Keywords:** Empathy, medical students, communication skills, clinical exposure, teaching

Dear Editor,

We were intrigued by Kim’s paper [1]. As senior medical students in the UK, it is our opinion that Medical Schools serve to enhance and maintain intrinsic empathy, as opposed to teaching an individual how to be empathetic [2]. We believe this is achieved in two fundamental ways: early patient interactions and teaching devoted to communication skills.

Patient contact is integral to our curriculums. From year 1, we conducted home visits on patients and were not limited for time during these encounters, enabling us to fully ascertain each individual’s biopsychosocial history. Dissimilar to Kim’s study, we were encouraged to engage with patients independently as opposed to in groups, which fosters a more personal, empathetic experience. Indeed, we firmly believe these experiences create a mutualistic relationship characterised by trust, where we may holistically understand the patient and refine our empathic skills.

Kim’s adoption of the IRI is an artificial measure of empathy, which problematises the evaluation of the true affective empathic experience. By contrast, our capacity for empathic intelligence was assessed prior to joining medical school as part of the selection process: we were shown staged videos of patient–doctor interactions and had to discuss ‘how the doctor demonstrated empathy’. From this, it is evident that empathy is not necessarily taught at Medical School. Rather, it is rather a prerequisite for admission, which develops cumulatively.

Our communication skills have been explored through interactive means. We were given a variety of clinical scenarios to role-play with simulated patients. These scenarios helped us to develop the verbal and nonverbal cues needed to enrich our communication skills. Moreover, we were required to critically appraise these performances, and this self-awareness facilitated the recognition of what skills we possessed or lacked in conveying empathy. Whilst this parallels the weekly seminars undertaken by the students in Kim’s study, our learning was/is not prescribed with the aim of completing an experiment and can therefore be perceived as more authentic. To this point: where Kim recognises the selection bias of interviewees in his study, we did not choose our scenarios. As such – in keeping with the medical profession – they encompassed a diverse range of individuals.

In conclusion, we believe that the capacity for empathy is innate and is a construct that may be appropriately matured through ones journey at Medical School. Our personal experiences of empathy range from observing the deterioration of a terminal cancer patient to sharing a patient’s excitement following the reversal of a Hartmann’s procedure. Critically, this first-hand, clinical mode of learning allows for an individualistic approach to developing empathy, which in turn provides society with unique, authentic doctors whose personable qualities are refined not through classroom teaching, but real-life interactions.

## References

[1] Kim KJ. Project-based learning approach to increase medical student empathy. Med Educ Online. 2020;25(1):1742965. 32197574 10.1080/10872981.2020.1742965 PMC7170272

[2] Batt-Rawden SA, Chisolm MS, Anton B, et al. Teaching empathy to medical students: an updated, systematic review. Acad Med. 2013;88(8):1171–1. 23807099 10.1097/ACM.0b013e318299f3e3

